# Malaria Night-Blindness

**Published:** 1884-03

**Authors:** 


### Micro-organisms in Water.

Osmic acid possesses the property of hardening protoplasm, hence microscopists make use of it for detecting animalcula in water. According to L. Maggi, the chloride of palladium may be substituted for the more poisonous osmic acid, an acid more dangerous than prussic acid. By adding to the water a solution of palladium chloride (one in eight hundred) he obtained a precipitate in which the bacterial forms could be recognized under the microscope just as distinctly as in those obtained with osmic acid.—*Gazz. Chim.*

### Malarial Night-blindness.

In the *Arch. of Ophthal.*, Zimmerman reports the case of a previously healthy family, who moved into a house in a low situation, close to a sewer. The children were attacked with intermittent fever and night-blindness. The fever yielded to quinine, but the visual defect presisted until removal to a healthy locality, when it vanished, never to return.

---

Mullein Plant in Phthisis.—It will be remembered that about one year ago we noted that Dr. F. J. B. Quinlan had recommended this article. In the *Brit. Med. Jour.* December 8, 1883, the same gentleman reports a case of pre-tubercular phthisis in which the patient gained twelve pounds in weight in one month, under the use of mullein. He considers that it possesses all the advantages and none of the drawbacks of cod liver oil.

---

A New Method of Tooth Drawing.—A small square of India-rubber, pierced with a central hole, is pushed over the tooth until the upper part of the root is reached. The rubber gradually contracts, pulls on the root, and the tooth is finally enucleated without causing any pain. Four or five days complete the operation, while very slight bleeding and slight swelling of the gum are the only inconveniences. This procedure is recommended by a dentist of Geneva.

---

The Physiological Action of Coffee.—In the *Brit. Med. Jour.*, we read that according to the result of experiments recently made by Messrs. Couty and Guimaraes, to ascertain the precise physiological action of coffee, that beverage is not a preventer of tissue-waste. The maintenance of nutrition is, no doubt, improved by its consumption, as Gubler asserted; but simply because it involves an increased assimilation of nitrogenous food through improving the appetite, when not taken in excess, and thereby encouraging its consumer to take nutritious food.

---

The study of histology ought to be insisted upon before permitting medical students to attempt the study of pathology; not by lectures alone, but by the use of the microscope in the laboratory.

